# A Cautionary Tale: Tn*903 aph*, Kanamycin Resistance Redux in the Environment

**DOI:** 10.1128/mBio.00392-17

**Published:** 2017-06-06

**Authors:** Vivian P. W. Miao

**Affiliations:** Department of Microbiology and Immunology, University of British Columbia, Vancouver, Canada; Johns Hopkins Bloomberg School of Public Health

**Keywords:** antibiotic resistance, commensal, environmental microbiology, resistome, transposons

## LETTER

The molecular biologist’s toolbox includes many engineered elements, such as plasmids, phages, and transposons, coupled to selectable markers such as genes conferring resistance to antibiotics. It is essential to prevent their escape from the laboratory so that they do not exacerbate the growing problem of antibiotic resistance in the environment (1, 2). Nonetheless, evidence for one occurrence was found among data originally collected by Wichmann et al. (3) as part of a functional metagenomics study of antibiotic resistance genes in uncultured bacteria in dairy cow manure. Fosmid DCM006Kan09, one of the clones associated with resistance to kanamycin in the study, was sequenced (GenBank accession no. KJ512980), and an *aph* gene, whose function was confirmed by subcloning and transformation of a sensitive host, was identified (3).

**FIG 1:**
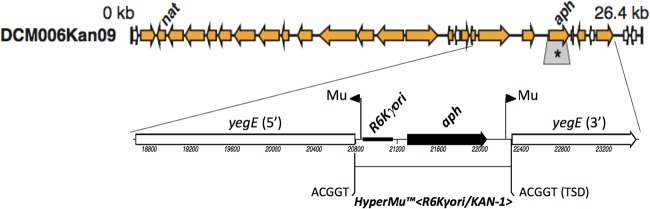
Fosmid DCM006Kan09 with details of *aph* region. Block arrows in the original map at the top (from Fig. 3 of reference 3) represent proteobacterial genes; *nat* and *aph* (boxed asterisk) indicate an *N*-acetyltransferase and an aminoglycoside phosphotransferase, respectively. The new, expanded diagram of the reannotated region around *aph* below the top map shows the HyperMu<R6K+ori/KAN-1> insertion in *yegE*, with a target site duplication (TSD) of nucleotides ACGGT from the upstream junction. Except for the transposon, TSD, and one missing nucleotide (from the fosmid), the metagenomic DNA cloned in DCM006Kan09 is identical to that of *E. coli* MG1655 (GenBank accession no. U00096.3; nucleotides 2124594 to 2149231).

Recent review of the sequences flanking *aph* reveals it to be part of the HyperMu<R6K+ori/KAN-1> transposon (EpiCentre) (Fig. 1). This element is characterized by an *aph*-carrying fragment from Tn*903* joined to the γ replication origin from plasmid R6K and bracketed at the termini by transposase recognition sequences (R1R2) for *Escherichia coli* phage Mu. Analysis of this part of DCM006Kan09 proceeded from first principles and was ultimately confirmed by comparison to the sequence of the transposon which, although not in GenBank (last search, 02 March 2017), was available from the manufacturer (http://www.epibio.com/tech-support/dna-sequences). In DCM006Kan09, this 1.5-kb element is inserted into DNA that otherwise has 99.9% continuous identity to sequences in numerous *E. coli* strains, e.g., MG1655 (GenBank accession no. U00096.3). The insertion disrupts the *yegE* gene and is accompanied by a 5-bp target site duplication, as is characteristic of phage Mu.

The recycling of *aph* from Tn*903*—housed in a different scaffold—into the environment via gut bacteria is an iatrogenic contribution to the evolution of mobile resistance determinants. While dedicated-purpose genetic constructs are often stripped down to “bare bones” and unable to easily propagate or relocate themselves, once integrated into a bacterial genome, such elements could replicate with the chromosome and be maintained. They might also become mobile if missing functions are provided in *trans* by indigenous wild-type elements in their host. Where a part of the construct may confer a selective advantage, e.g., *aph*, even minimal elements have the potential to persist and become part of the environmental resistome.

The presence of a synthetic transposon in an environmental data set is a cautionary reminder. Researchers have the responsibility not only to monitor but also to reduce proliferation of antibiotic resistance, and both designers and users have opportunities for updating and safeguarding communal tools involving resistance genes.
